# Moral Thinking and Empathy in Cognitive Behavioral Therapy for Children and Adolescents with Conduct Problems: A Narrative Review

**DOI:** 10.1007/s10567-023-00429-4

**Published:** 2023-03-11

**Authors:** Walter Matthys, Dennis J. L. G. Schutter

**Affiliations:** 1 Department of Clinical Child and Family Studies, Utrecht University, Heidelberglaan 1, 3584 CS Utrecht, The Netherlands; 2 Department of Experimental Psychology, Helmholtz Institute, Utrecht University, Heidelberglaan 1, 3584 CS Utrecht, The Netherlands

**Keywords:** Cognitive behavioral therapy, Conduct problems, Moral thinking, Empathy, Children, Adolescents

## Abstract

Cognitive behavioral therapy (CBT) for conduct problems in children and adolescents aims to decrease behaviors which may be considered moral transgressions (e.g., aggressive and antisocial behavior) and to increase behaviors that benefit others (e.g., helping, comforting). However, the moral aspects underlying these behaviors have received relatively little attention. In view of increasing the effectiveness of CBT for conduct problems, insights into morality and empathy based on studies from developmental psychology and cognitive neuroscience are reviewed and integrated into a previously proposed model of social problem-solving (Matthys & Schutter, Clin Child Fam Psychol Rev 25:552–572, 2022). Specifically, this narrative review discusses developmental psychology studies on normative beliefs in support of aggression and antisocial behavior, clarification of goals, and empathy. These studies are complemented by cognitive neuroscience research on harm perception and moral thinking, harm perception and empathy, others’ beliefs and intentions, and response outcome learning and decision-making. A functional integration of moral thinking and empathy into social problem-solving in group CBT may contribute to the acceptance of morality-related issues by children and adolescents with conduct problems.

Children and adolescents with clinical levels of conduct problems either meet criteria of oppositional defiant disorder or conduct disorder (American Psychiatric Association, 2013) or show symptoms in the clinical range of defiant behavior, irritability, aggressive behavior or antisocial behavior on a standardized measure of psychopathology (e.g., the Achenbach System of Empirically Based Assessment (ASEBA); Achenbach, 2009). According to the latest large meta-analysis on the effectiveness of psychological therapy for children and adolescents treated for mental health problems in the clinical range, the mean post-treatment effect size (ES, Cohen’s *d*) for conduct problems is 0.46 (Weisz et al., 2017). Notably, the mean effect size of psychotherapy for conduct problems in children and adolescents has been shown to decrease over the last 50 years (1963–2016), suggesting that psychotherapy could benefit from amending some of the approaches that have been used thus far (Weisz et al., 2019).

The need for amending treatment approaches may also hold for cognitive behavioral therapy (CBT) with a mean ES of 0.35 as observed in the meta-analysis by McCart et al. (2006). This meta-analysis found a positive relationship between age and ES, showing that as youth ages and progresses into more advanced levels of cognitive development they benefit more from CBT. Importantly, however, in the meta-analysis by Armelius and Andreassen (2007), the mean ES of CBT in youths aged 12–22 for the treatment of antisocial behavior in secure or non-secure residential settings is 0.25. Clearly, attempts at increasing effectiveness of CBT as a psychological treatment for conduct problems in middle childhood and adolescence are needed. Incidentally, CBT is not offered as a stand-alone treatment for conduct problems, but combined with behavioral parent training in childhood (Kazdin et al., 1992; Zonnevylle-Bender et al., 2007) and family-based psychotherapy in adolescence (Alexander et al., 2013), or is part of an intervention targeting multiple systems in adolescence (Henggeler et al., 2009). In CBT children and adolescents learn social problem-solving skills that enable them to behave in more independent and situation appropriate ways (Matthys & Schutter, 2022).

Modifications of CBT programs may be needed as most evidence-based CBT programs were developed during the last three decades of the previous century, undergoing only modest updates in more recent years. CBT teaches children from 7 years on and adolescents social problem-solving and anger regulation skills. Social problem-solving, anger management, and affect education (i.e., understanding, identifying, and labeling emotions) are common core therapeutic elements of evidence-based practice for children with disruptive behavior problems (Garland et al., 2008). Notably, moral thinking and empathy do not standardly belong to the common core therapeutic elements for children aged 4 to 13 with conduct problems (Garland et al., 2008).

Likewise, a review of evidence-based psychosocial treatments for adolescents with disruptive behavior shows that improving moral thinking in CBT is typically not part of conventional CBT (McCart & Sheidow, 2016). Yet there are some treatment programs that include sessions aimed at improving moral reasoning such as EQUIP (Gibbs et al., 1995). Based on the positive findings from the initial randomized controlled trial (Leeman et al., 1993), EQUIP meets criteria as a probably efficacious treatment for disruptive adolescents detained in correctional facilities (McCart & Sheidow, 2016). The sessions in EQUIP (Gibbs et al., 1995) aimed at improving moral reasoning are guided by Aggression Replacement Training (Glick & Gibbs, 2011). However, with regard to Aggression Replacement Training, results from a systematic review indicate that there is insufficient evidence to substantiate the hypothesis that Aggression Replacement Training has a positive impact on recidivism, self-control, social skills or moral development in adolescents and adults (Brännström et al., 2016). Finally, Moral Reconation Therapy, developed for the treatment of adult and adolescent offenders, also includes sessions aimed at improving moral reasoning (Little & Robinson, 1988). In a meta-analysis of Moral Reconation Therapy criminal offending subsequent to treatment was the outcome variable (Ferguson & Wormith, 2012). The overall effect size (*r* = 0.16) of 33 studies indicated that Moral Reconation Therapy had a small positive effect on recidivism. Youth, however, benefited from Moral Reconation Therapy less than adults (Ferguson & Wormith, 2012). Importantly, in comparison to the theoretical approaches of moral reasoning underlying the treatment programs, alternative theories of morality have been developed over the last decades (see section on Developmental Psychology).

It is noteworthy to mention that in CBT for conduct problems little attention is paid to moral functioning while characteristic behaviors (e.g., aggression, antisocial behavior) may be considered moral transgressions. Morality, on the one hand, involves the acquisition by individuals of societal and cultural values, but, on the other hand, involves principles and obligations that go beyond the identification of societal and cultural values, such as those related to inflicting pain and harm on others (Turiel, 2023). Morality not only deals with norms prohibiting behaviors that harm others but also with norms benefiting others, that is prosocial behaviors (e.g., helping, sharing, comforting).

In this context, empathy is also relevant for CBT in treating conduct problems as empathy may be a motivator for prosocial behavior. Empathy is an affective response that stems from the apprehension or comprehension of another’s emotional state and is similar to what the other person is feeling (Spinrad et al., 2023). Available school-based interventions for children and adolescents which promote empathy-related responding have shown to have a small but positive effect on conduct problems (*d* = 0.17) (Malti et al., 2016).

The aim of the present narrative review is to position moral thinking and empathy in our previously described model of social problem-solving for conduct problems consisting of the following psychological skills: (1) recognition of problematic social situations, (2) recognition of facial expressions, (3) emotion awareness and regulation, (4) behavioral inhibition and working memory, (5) interpretation of the social problem, (6) affective empathy, (7) generating appropriate solutions, (8) evaluations of solutions based on outcome expectations and moral beliefs, and (9) decision-making (Matthys & Schutter, 2022). Our model is based on D’Zurilla and Goldfried’s (1971) model of problem-solving and the Crick and Dodge (1994) model of social information-processing. In view of integrating morality in our model of social problem-solving, we first review studies on moral thinking and empathy in children and adolescents with conduct problems (or with high levels of aggressive behavior which are not clinically defined), from both the perspective of developmental psychology and cognitive neuroscience. Next, implications of results for CBT based on the model of social problem-solving by Matthys and Schutter (2022) will be discussed.

From here on we use the term children for both children and adolescents, except in studies of adolescents and when making statements about adolescence specifically.

## Developmental Psychology

### Social Domain Theory

Lawrence Kohlberg was the first to articulate a developmental theory of morality based on interviews to assess children’s and adolescents’ moral reasoning (Colby et al., 1983). According to this global cognitive-developmental theory, youth’s moral judgement develops through a series of qualitative distinct phases (Colby et al., 1983). However, researchers have criticized Kohlberg’s use of complex hypothetical vignettes to study morality. Alternatively, children’s judgements were studied in response to hypothetical stories in different domains of social knowledge. Studies have shown that children distinguish moral issues (e.g., issues of justice and rights) from conventional rules (e.g., how to address a teacher) at earlier ages than Kohlberg’s theory suggests (e.g., Smetana, 1989). According to this social domain perspective on moral development (i.e., social domain theory of moral development) the variety of social experiences is essential to an understanding of children’s moral development (Turiel, 1998).

Specifically relevant for children with conduct problems are their characteristic experiences as initiators, perpetrators, observers, and victims of moral transgressions. Arguably due to temperamental and atypical neurobiological characteristics from an early age on these children can elicit coercive interactions in their parents, some of whom live in stressful family environments and have limited personal resources themselves due to mental health problems and marital discord (Patterson, 2002; Stormshak et al., 2018).

Coercive cycles of interactions between children and their parents, among their parents, with their siblings, and with their peers can lead to moral transgressions such as verbal and physical aggression. These social experiences arguably contribute to the development of moral thinking in these children and adolescents. According to cognitive psychologists experiences are stored in long-term memory and these traces are integrated to form schemas (Fiske & Taylor, 1991). These schemas simplify the cognitive tasks involved in information-processing, such as those in social information-processing as described by Crick and Dodge (1994): (1) encoding of social cues, (2) interpretation of those cues, (3) clarification of goals, (4) search of potential behavioral responses, (5) decision of the behavioral response based on moral evaluation of responses and outcome expectations, and (6) the behavioral enactment of that response. Thus, children’s social experiences with moral transgressions stored in schemas affect social information-processing (see Arsenio and Lemerise (2004) who were the first to present an integration of social domain theory of moral development and social information-processing theory). We next discuss how both normative beliefs about aggression and goal orientations stored in schemas may affect social information-processing, as described by Crick and Dodge (1994), in children with conduct problems or with high levels of aggression which are not clinically defined.

### Normative Beliefs in Support of Aggression and Antisocial Behavior

Huesmann and Guerra (1997) introduced the concept normative beliefs in psychology, defined as the individual’s cognitive standards about the acceptability or unacceptability of a behavior; children and adolescents acquire normative beliefs through observation, experience, and tuition they receive from peers, parents, and teachers. Huesmann and Guerra (1997) found that in fourth and fifth graders individual differences in normative beliefs that aggressive forms of behavior are socially acceptable and appropriate, lead to an increase in aggressive behavior as sixth graders.

Huesmann and Guerra (1997) hypothesized three ways in which normative beliefs affect children’s aggressive behavior. First, normative beliefs may affect the way in which children perceive or interpret the behaviors of others; the more children approve of aggression, the more likely they may be to perceive hostility in others, even if no hostility is present (step 2 in the social information-processing model). Second, normative beliefs in support of aggression may cue the retrieval of aggressive scripts for social behavior. In other words, normative beliefs may help generating aggressive solutions to social problems (step 4). Finally, if normative beliefs act as filters to eliminate "inappropriate" behaviors from children's repertoires, children with normative beliefs in support of aggression are less likely to reject aggressive solutions once they have thought of them as solutions to social problems. Thus, normative beliefs may play a role in the evaluation step of social information-processing (step 5). The hypotheses by Huesmann and Guerra were confirmed in a study by Zelli et al. (1999). Individual differences in retaliation approval among third graders predicted individual differences in fifth graders’ aggressive behavior; nearly 50% of this effect could be attributed to three social information-processing steps: (1) attribution of hostile intentions, (2) generation of aggressive responses, and (3) positive evaluation of aggressive responses (Zelli et al., 1999).

In addition, positive evaluation of aggressive behavior (step 5), including social acceptability and moral appropriateness of aggression, incremented the prediction from externalizing behavior in early adolescence to later antisocial problems (Fontaine et al., 2002). The distinction between reactive and proactive aggression may be relevant here. Controlling for reactive aggression, higher levels of proactive aggression in adolescents were associated with lower moral concerns (i.e., deny or minimize negative consequences for others) regarding one’s aggression (Arsenio et al., 2009). There is also evidence that atypical outcome expectations and normative or moral beliefs (step 5) are related to callous-unemotional traits or limited prosocial emotions (i.e., lack of empathy, lack of remorse or guilt, shallow or deficient affect, and unconcerned about performance). In a study with adjudicated youth, higher callous-unemotional traits were related to increased expectations and values associated with the positive consequences of aggression (i.e., tangible rewards, dominance) and decreased expectations and values associated with the negative consequences of deviant behavior (i.e., punishment) (Pardini et al., 2003).

The implications of the findings on normative beliefs for CBT are discussed in the section Moral Thinking and Empathy in CBT, specifically in Step 5 Interpretation and empathy, Step 7 Generation of Solutions, and Step 8 Evaluations of solutions based on outcome expectations and normative beliefs (see also Table 1).

**Table 1:** What children and adolescents learn in cognitive behavioral therapy

Step	Psychological skill
1	Recognition of problematic social situations
	Which social situations are problematic for me? This is important to know in view of starting thinking in a situation that might be difficult for me to handle
2	Recognition of facial expressions
	What do other person’s facial expressions tell me about their feelings and about a possible social problem? If the other person feels anxious, sad, or angry this person can expect me to respond to this. I really need to think now
3	Emotional awareness and regulation
	What do I feel myself? And in case my own feeling (e.g., anxiety, sadness, anger) is too strong, what can I do to cope with this feeling?
4	Behavioral inhibition and working memory
	I shouldn’t act right away. Rather, I should think first and concentrate on what is the problem and how to solve it
5	Interpretation and empathy
	When I see that someone else has done something bad to me, I shouldn’t always think this person did that on purpose. Maybe I’m inclined to think this because I’m convinced that’s the way how people treat each other. On the other hand, when I see that the other person feels bad I should try to understand what did happen. Maybe I don’t see that I did hurt the other because I’m not used to paying attention to it. And what do I feel myself when I see how this person feels? Do I understand what this person might feel? Am I prepared to care for this person?
6	Clarification of goals
	When I want to solve this problem, what is my goal? If I know my goal, I can better think about what to do. Do I want to get my way or revenge for wrongs done? Or do I want to work things out and find a solution together with this person?
7	Generation of solutions
	In a difficult situation I must try to come up with one or more solutions. Maybe I think that only aggressive solutions work. Perhaps there are also solutions that bring both me and the other person benefits? To find such ‘kind’ solutions, I need to think what the problem actually is (step 5) and what I want to achieve (step 6)
8	Evaluation of solutions based on outcome expectations and normative beliefs
	Then, I would do well to think about consequences of solutions for me, the other person, and our relationship, both on the short and long-term. I may have difficulty believing that positive consequences can also be expected to result from solutions that are clearly not aggressive but ‘kind’ (or constructive) instead. Related to this, I would do well to consider if the solutions I’m thinking about do not harm the other person
9	Decision-making
	In the end I choose the best solution for both of us. For this, I have to make connections between previous steps: What is the problem? What is my goal? What are possible solutions? And what is the solution that is most beneficial for the other person and myself? This can be difficult for me. But many positive experiences with appropriate solutions will help me making correct decisions

### Clarification of Goals

Moral thinking can also affect social information-processing in the clarification of goals step (step 3). Goals function as orientations toward producing particular outcomes and are thought to influence subsequent response generation (Crick & Dodge, 1994). Moral values (i.e., what is believed to be good and what should or should not be done) stored in latent mental structures or schemas may affect goal orientations. For example, among adolescent boys a consistent association has been reported between high goal values for dominance and revenge, and low values for affiliation on the one hand, and a wide range of delinquent, substance-using, and behavioral difficulties on the other hand. Dominance proved to be the most sensitive correlate of these negative outcomes (Lochman et al., 1993). In another study, 9- to 12-year-old proactive-aggressive children were less likely than their peers to endorse relationship-enhancing goals during social interaction. Rather, they were more likely to prefer goals that are instrumental in nature (Crick & Dodge, 1996).

Among the sources of goal orientations Crick and Dodge (1994) mention feelings. For example, feeling angry might serve as an impetus for a retaliatory goal. In addition, the intensity with which children experience emotions and their emotion regulation capacities are relevant to mention here. Children who are overwhelmed by emotions may choose hostile goals to reduce their own arousal (Lemerise & Arsenio, 2000). In a longitudinal study of 4th through 7th graders three revenge goal trajectory groups were identified: a low-stable group, an increasing group, and a decreasing group (McDonald & Lochman, 2012). Aggressive children who found it difficult to regulate their angry and anxious emotions and inhibit their behaviors endorsed revenge goals at initially higher levels and continued to endorse revenge at higher levels for at least two more years (McDonald & Lochman, 2012).

The implications of the findings on clarification of goals for CBT are discussed in the section Moral Thinking and Empathy in CBT, in particular in Step 6 Clarification of goals and Step 7 Generation of solutions (see also Table 1).

### Empathy

Empathy can be defined as an affective response that stems from the apprehension or comprehension of another’s emotional state and is similar to what the other person is feeling (Spinrad et al., 2023). A distinction has been made between affective and cognitive empathy. Cognitive empathy refers to the recognition and understanding of another’s experience, whereas affective empathy refers to the ability to share another’s emotion (Spinrad et al., 2023). Further, sympathy is distinguished from empathy as sympathy consists of feelings of sorrow or concern for the other (Spinrad et al., 2023). Sympathy is an emotional response stemming from the apprehension of another’s emotional state, but it does not per se involve experiencing the same emotion as the other would be expected to experience (Spinrad et al., 2023). In other words, sympathy is feeling *for* and empathy is feeling *with* (Colasante et al., 2023). Finally, empathy can lead to personal distress resulting in an orientation towards the self which interferes with attending to the needs of others (Spinrad et al., 2023).

In research on the relation between empathy and behavior these distinctions often are not made (Spinrad et al., 2023). Empathy and sympathy may be expected to be associated with prosocial behavior. Eisenberg and Miller (1987) reported low to moderate positive correlations (between 0.10 and 0.36) between empathy and both prosocial behavior and cooperative/socially competent behavior. More recent studies have confirmed the relation between empathy and sympathy with measures of social competence and adjustment (Spinrad et al., 2023). Furthermore, a negative relation of empathy with aggression may be expected. In support of this assumption, Miller and Eisenberg (1988) found low-to-moderate negative correlations (between − 0.06 and − 0.46) between empathy and aggression, externalizing and antisocial behaviors, and enactment and receipt of physical abuse. In addition, Zuffianò et al. (2018) found evidence for a co-developmental process linking sympathy and aggression in children aged 6 to 12 years, suggesting that improvements in sympathy were linked to declines in aggression and vice versa. Likewise, in a longitudinal study with adolescents higher levels of sympathy were found to mitigate future aggressive behaviors (Carlo et al., 2010).

In addition to these developmental psychology studies on the relations between empathy and aggressive or antisocial behavior, studies on empathy have also been performed among children with conduct problems. For example, using empathy-inducing video-vignettes deficits in empathy have been observed in 8- to 12-year-old boys with conduct problems when compared to typically developing boys (De Wied et al., 2005). Also, using a computer task 6 to 7-year old children with conduct problems showed less empathy-induced prosocial behavior in response to sadness and distress compared to typically developing children (Deschamps et al., 2015). Psychophysiological approaches such as facial mimicry and heart rate have also been used. Facial mimicry is a component in the process of empathy whereas exposure to sadness is associated with heart rate deceleration. Relative to controls, boys with conduct problems showed less heart rate reduction during sadness and smaller increase in corrugator electromyographic (EMG) activity (i.e., frowning) to sadness and angry facial expressions (De Wied et al., 2009). Likewise, male adolescents with conduct problems and high callous-unemotional traits showed less corrugator EMG activity and less heart rate change from baseline during exposure to empathy-inducing film clips portraying sadness as compared to controls (De Wied et al., 2012).

The implications of the findings on empathy for CBT are discussed in the section Moral Thinking and Empathy in CBT, in particular in Step 3 Emotion awareness and regulation, Step 5 Interpretation and empathy, and Step 7 Generation of solutions (see also Table 1).

## Cognitive Neuroscience

### Harm Perception and Moral Thinking

From the beginning of this century neuroimaging studies have been conducted investigating brain correlates of psychological functions including those involved in the processing of moral norms. Care-based norms are specifically relevant for conduct problems. Care-base norms are standards regarding actions that might harm others, including the theft or damage of others’ property (Blair, 2023). Care-based morality refers to those forms of moral reasoning that concerns actions that harm others (Blair, 2007).

Stimulus-reinforcement learning allows healthy individuals to learn the value of a stimulus (Blair, 2017). In particular, Blair (2017) argues that one’s sense of “badness” of care-based moral transgressions is associated with an aversive unconditioned stimulus, that is the distress of the other individual. Accordingly, typically individuals learn to avoid actions associated with another individual’s distress. Impairments in stimulus-reinforcement learning and in responsiveness to the distress of other individuals disrupt individual’s ability to learn the emotion-based sense of badness of care-based moral transgressions (Blair, 2017).

The neural circuitry underlying stimulus-reinforcement learning includes the amygdala where information about the conditioned stimulus and unconditioned stimulus converge (Blair, 2017). Reduced amygdala responsiveness to the distress of other individuals has been shown in children with conduct problems and callous-unemotional traits (Marsh et al., 2008). In addition to the amygdala other regions that are involved in stimulus-reinforcement learning include the hippocampus, ventromedial prefrontal cortex and the dorsal anterior cingulate cortex (Blair, 2017). Care-based judgements rely, first, on the amygdala associating the aversive emotional response to the victim’s distress with the representation of the action that caused this distress, and, second, on the ventromedial prefrontal cortex representing the value of the transgression (Blair, 2007). In agreement, adolescents with conduct problems and psychopathic traits showed reduced amygdala activity and lower-than-normal functional connectivity between the amygdala and orbitofrontal cortex when making judgements about legal/illegal actions (Marsh et al., 2011). The authors suggest that psychopathic traits may affect adolescents’ ability to attach the appropriate affective valence to actions of varying moral permissibility, and from using information about valence to guide their decisions (Marsh et al., 2011). Thus, deviances in perception of harm and moral transgressions occur in children and adolescents with conduct problems. These are relevant for the interpretation step in Crick and Dodge’s social information-processing model (1994).

The implications of the findings on harm perception and moral thinking for CBT are discussed in the section Moral Thinking and Empathy in CBT, in particular in Step 5 Interpretation and empathy, Step 7 Generation of solutions, and Step 8 Evaluation of solutions based on outcome expectations and normative beliefs (see also Table 1).

### Harm Perception and Empathy

Perceiving others being harmed not only initiates moral thinking but elicits empathic concern as well (Decety & Cowell, 2018). Blair (1995) suggested that in humans a victim’s pain and distress induce similar feelings of distress in the aggressor, which in turn stops further aggressive behavior. Empathy may be considered a multidimensional construct comprising dissociable components that interact and operate in parallel fashion, including affective, motivational, and cognitive components (Decety & Cowell, 2014; Decety & Jackson, 2004). Three components may be distinguished: (1) the emotional component reflecting the capacity to share or become affectively aroused by others’ emotions; (2) the motivational component of empathy (empathic concern) corresponding to the urge of caring for another’s welfare; (3) the cognitive component which is similar to the construct of perspective taking (Decety & Cowell, 2014; Decety & Jackson, 2004). Functional neuroimaging studies using pain perception tasks have shown neural activation in a network of regions comprised of the amygdala, anterior insula cortex and anterior cingulate cortex (see Blair et al., 2018;Yoder et al., 2016).

Several functional neuroimaging studies have shown deficiencies in affective empathy among children with conduct problems. In a study among adolescents with conduct problems and psychopathic traits activation in brain structures associated with empathic pain perception was assessed. To this end, adolescents viewed photographs of pain-inducing injuries and were instructed to imagine either that the body in each photograph was their own or that it belonged to another person (Marsh et al., 2013). Adolescents with conduct problems showed reduced activity in regions associated with affective responses to other’s pain as the depicted pain increased which included the rostral anterior cingulate cortex, ventral striatum, and amygdala (Marsh et al., 2013). In addition, these youths showed a lack of response of the amygdala and insular cortex to increases in other’s pain, but not own pain (Marsh et al., 2013). In another study, reduced activity in the insular cortex while viewing others being harmed was related to children’s greater conduct problem symptoms and callousness (Michalska et al., 2016). Also, among children with conduct problems those with higher callous traits showed less functional connectivity seeded in anterior cingulate with left amygdala and anterior insular cortex when they were exposed to visual stimuli depicting other people being physically injured (Yoder et al., 2016). The decreased connectivity suggests that children with high callous traits encode the pain of others as less salient than children with low callous traits (Yoder et al., 2016).

The implications of the findings on empathy for CBT are discussed in the section Moral Thinking and Empathy in CBT, in particular in Step 3 Emotional awareness and regulation, Step 5 Interpretation and empathy, and Step 7 Generation of solutions (see also Table 1).

### Others’ Beliefs and Intentions

Representations of others’ beliefs and intentions (i.e., social cognitions, mental state understanding, theory of mind) are relevant in moral reasoning and are part of the interpretation step in the social-information processing model (Crick & Dodge, 1994). The posterior superior temporal sulcus, temporoparietal junction and medial prefrontal cortex are essential regions for incorporating intentionality into moral judgments (Yoder & Decety, 2018). For example, transient disruption of the right temporoparietal junction using transcranial magnetic stimulation causes people to judge attempted harms as less morally forbidden and more morally permissible (Young et al., 2010). In adults, this differentiation between intentional and accidental harm in the right temporoparietal junction occurs within less than one-tenth of a second after the stimulus is perceived (Decety & Cacioppo, 2012). Thus, information about intent is rapidly integrated into harm-based moral judgements (Rottman & Young, 2015).

A functional magnetic resonance imaging study showed that when antisocial adolescents performed the mini-ultimatum game, they took the intention of the other player less into account and this was associated with less activation in the temporoparietal junction (Van den Bos et al., 2014). In ultimatum games one player, the proposer, is endowed with a sum of money. The proposer is tasked with splitting it with another player, the responder. Once the proposer communicates the decision, the responder may accept or reject the offer. If the responder accepts, the money is split per the proposal, but if the responder rejects, both players receive nothing. In this version of the ultimatum game a single unfair offer is presented together with an alternative offer (fair alternative, no alternative, hyperfair alternative). Adolescents rejected unfair offers significantly less when the proposer presented no alternative offer as compared to when proposer presented a fair or hyperfair offer. This result suggests that the adolescents took the intentions of the proposer into account. However, this effect was reduced in antisocial adolescents. Indeed, antisocial adolescents show less acceptance of the no-alternative condition compared with the control group. This indicates that they react more strongly to the unfairness of the offer and are less concerned about the intentions of the proposer. According to the authors, this finding agrees with studies that found perspective-taking skills are not necessarily deficient in these adolescents, but they may not spontaneously engage them (Van den Bos et al., 2014).

In another study, the neural correlates of fairness decisions in response to communicated emotions of others were examined in a sample of criminal-justice involved boys with conduct problems (Klapwijk et al., 2016). Results showed that the boys with conduct problems compared with typically developing boys had less activity in the right temporoparietal junction and supramarginal gyrus when receiving happy in contrast to disappointed and angry reactions. The authors suggest that decreased activation of these brain areas in the conduct problems group indicate that boys with conduct problems are less inclined to take the perspective of the other person displaying positive as compared to negative reactions (Klapwijk et al., 2016). In line with this reasoning are study results showing that adult psychopathic individuals have a diminished propensity to automatically think from another’s perspective (Drayton et al., 2018). If individuals are less inclined to take the perspective of others they are probably at risk of difficulty distinguishing incidental from intended harm by others and, as a result, formulating revenge goals.

The implications of the findings on Others’ beliefs and intentions for CBT are discussed in the section Moral Thinking and Empathy in CBT, specifically in Step 5 Interpretation and empathy (see also Table 1).

### Response Outcome Learning and Decision-Making

Response decision is the final step in the social information-processing model, preceding behavioral enactment (Crick & Dodge, 1994). Appropriate decision-making is based on response outcome-learning which allows the individual to represent the value of committing a particular action (Blair, 2017). A core feature of moral judgement is considering the expected value of the moral or immoral action (Blair, 2017). Disrupted representation of the expected value is thought to mean that the individual is less likely to avoid actions that harm others (Blair, 2017). Difficulties in decision-making based on uncertainties about positive and negative outcomes can impede children’s ability to make decisions about appropriate solutions to social problems.

Reinforcement-based decision-making studies show that reduced neural responsiveness to reward puts an individual at risk of poor decision-making because response choices are less guided by expectations that an action will result in reward relative to punishment (Blair et al., 2018). A meta-analysis of whole-brain fMRI studies showed that the most consistent dysfunction in children with conduct problems involves the rostro-dorsomedial, fronto-cingulate and ventral-striatal regions that mediate reward-based decision-making (Alegria et al., 2016). With regard to punishment-based decision-making, the anterior insular cortex, dorsomedial frontal cortex and caudate nucleus of the striatum have been found to be implicated in avoidance-related behavior (Blair et al., 2018). Dysfunctions in these regions when making suboptimal choices as a function of expected value have been found in adolescents with conduct problems (White et al., 2014) and are correlated to increased risk for antisocial behavior (White et al., 2016).

The implications of the findings on Response outcome learning and decision-making are discussed in the section Moral thinking and empathy in CBT, in particular in Step 9 Decision-making (see also Table 1).

## Moral Thinking and Empathy in CBT

To examine the possible implications of moral thinking and empathy for CBT we further developed the social problem-solving model by Matthys and Schutter (2022) and present an overview of this adapted model in Table 1. Analogous to the Crick and Dodge model (1994), the model consists of nine steps. The first four steps are crucial in view of starting up the social problem-solving process and preventing the disruption of this process; these steps are only briefly discussed here (for more details see Matthys & Schutter, 2022). Empathy, however, is an important theme in Step 3. From Step 5 on we extensively describe the role of moral thinking and empathy in the interpretation step (Step 5), clarification of goals (Step 6), generation of solutions (Step 7), evaluation of solutions (Step 8), and decision-making (Step 9).

Conduct problems are heterogeneous in nature. Children with conduct problems not only differ in typical symptoms (e.g., defiant behavior, irritability, aggressive behavior, antisocial behavior, limited prosocial emotions), but also in symptoms of associated conditions (e.g., attention problems, impulsivity, depressive mood, anxiety, deficits in intellectual functioning). Related to this, when CBT is offered to a group of children with conduct problems then this group will most likely consist of children with weakly developed but also with well-developed psychological functions or skills. For this reason, CBT needs to be tailored to the child’ characteristics of psychological skills; this also applies when CBT is offered individually. This variety in psychological skills can be used for therapeutic purposes. For example, in a session on a specific psychological skill, a child with a reasonably well developed psychological skill can act as a model for others whose psychological skill is less well developed. Likewise, the variety of normative beliefs about the acceptability of oppositional, antisocial and aggressive behaviors is used to elicit discussions in view of changing these beliefs. Importantly, parents and other adults including teachers, and child care workers in day treatment, inpatient treatment, and residential treatment are also involved in CBT to elicit, support, and reinforce children’s use of the psychological skills in everyday life (in vivo practice).

In *step 1*, children learn which particular situations are challenging for them, such as being provoked by a peer or being expected to comfort a peer who is troubled, worried or upset (Dodge et al., 1985; Matthys et al., 2001; Van der Helm et al., 2013). Importantly, how children with conduct problems solve social problems depends on the types of social problems (Matthys et al., 1999; Van Rest et al., 2020). In addition, knowing which particular situation is problematic is important for the child in view of starting social problem-solving activity. After all, social problem-solving in a step-by-step manner is anything but ordinary for children. Issue of morality may be introduced here in terms of finding solutions which are beneficial both for the child and the peer or adult involved in the problematic social situation. For this step and all subsequent steps, psychotherapists can use written scenarios of problem situations, videos depicting a range of problem situations, and children’s own experiences in problem situations.

*Step 2* is about face recognition. Facial expressions of others have a communicatory function as they signal relevant information, such as feelings and intentions, to the observer (Blair, 2003). We suggest that recognition of other’s facial expressions sets in motion the social problem-solving process. Meta-analyses have shown impaired emotion recognition in children with antisocial behavior (Marsh & Blair, 2008) or with psychopathic (callous-unemotional) traits (Dawel et al., 2012). If displays of fear, sadness or anger are not recognized then there is a risk that a potential social problem is ignored and social problem-solving activity is not started. Improving facial emotion recognition is therefore crucial. There is some evidence that facial emotion recognition can be changed. Children with disruptive behavior referred to a program to prevent antisocial outcomes and who showed impairments in facial emotion recognition completed a computerized intervention designed to improve the identification of facial expressions. Children improved significantly in recognition of sadness, fear, anger and neutral facial expressions (Hunnikin et al., 2022).

*Step 3* involves becoming aware of one’s own emotions elicited by the social problem and regulation of one’s own emotions. Becoming aware of one’s own emotions is also relevant for empathy, as the capacity to become affectively aroused by others’ emotions is the first component of empathy (Decety & Cowell, 2014; Decety & Jackson, 2004). Low emotional awareness (i.e., difficulty identifying and labeling one’s emotions) has been shown to be associated with psychopathology, including aggression and rule-breaking in children (Weissman et al., 2020). Following emotion awareness, emotions may need to be regulated. Female adolescents with conduct problems have been found to be less successful than typically developing adolescents in emotion regulation by cognitive reappraisal (Raschle et al., 2019). In CBT children first learn to identify physiological cues of anger (e.g., hot flushes, faster heart rate, tightened muscles) as well as find words for various levels of anger (e.g., irritated, mad, furious) with the use of an anger thermometer (Lochman et al., 2008). They then learn to use coping self-statements (i.e., cognitive reappraisal), distraction techniques, and brief deep-breathing relaxation methods to handle the arousal associated with anger (Lochman et al., 2008). Learning to handle the arousal aspect of emotions is also relevant for empathic responding as empathy can lead to personal distress resulting in an orientation towards the self which interferes with attending to others’ needs (Spinrad et al., 2023).

In *step 4,* CBT therapists work with children on behavioral inhibition and working memory problems. Impairments in the ability to inhibit impulses can prevent children with conduct problems from starting the thinking process before acting, especially those with symptoms (or full diagnosis) of attention-deficit/hyperactivity disorder (see Matthys & Schutter, 2022). Children learn not to act right away in a problem situation, but think first and concentrate on the nature of the problem. In addition, working memory may be relevant for social problem-solving in children with conduct problems, especially those with attention-deficit/hyperactivity disorder diagnosis or symptoms (see Matthys & Schutter, 2022). Working memory arguably affects interpretation (step 5) as interpretation involves assembling multiple pieces of potentially contradictory information. Results of recent studies showing positive effects of central executive training targeting working memory on response inhibition and hyperactivity are promising (Kofler et al., 2018, 2020). Thus, for some children with severe executive function deficits executive training may be useful.

*Step 5* involves the interpretation of the social problem, including empathy. Hostile attribution biases or the tendency to attribute hostile intent to peers in social situations with a negative outcome have been demonstrated in aggressive children (De Castro et al., 2002; Verhoef et al., 2019). Therefore, in CBT therapists work on children’s perspective taking abilities to correctly infer the other’s intentions and thoughts. There is also evidence that adolescents with conduct problems are less inclined to take the perspective of the other person (Klapwijk et al., 2016; Van den Bos et al., 2014). Thus, in addition to improving perspective taking abilities, attentional focus is also needed to the propensity to think from another’s perspective, for example, using role-playing.

In addition, normative beliefs as cognitive standards about the acceptability or unacceptability of aggression and antisocial behavior are important as well, as they may affect the way children perceive (or interpret) the behaviors of others. In particular, the more children approve of aggression, the more likely they may be to perceive hostility in others, even if no hostility is present (Huesman & Guerra, 1997; Zelli et al., 1999). In other words, when they have been treated unfairly, they shouldn’t by default think that this was done on purpose. They should come to understand that this is because they think this is the way how people treat each other. Changing normative beliefs in support of aggression may result in decrease of hostile intent attributions. Thus, while working on hostile intentions normative beliefs in support of aggression should be an important topic.

In the interpretation step, CBT therapists may also want to pay attention to children’s difficulties perceiving other’s being harmed. As a result, these difficulties lead to reduced care-based judgements. Indeed, some children with conduct problems, perhaps specifically those with limited prosocial emotions, have deviant care-based norms (i.e., norms concerning actions that can harm others physically or psychologically, including the theft or damage of others’ property) (Blair, 2023). As a result, when they are involved in a conflict with a peer, the conflict threatens to escalate, because they do not pay attention to the damage they are inflicting on their peer. Sharing and discussing care-based norms among children with different types of care-based judgements may help perceiving harm and generate conciliatory behavioral solutions to be used in the midst of conflicts. Sharing and discussing care-based norms may also prevent children with conduct problems from harming others physically or psychologically outside the context of conflicts.

Perceiving others being harmed not only initiates moral thinking (care-based judgements) but empathic concern as well (Decety & Cowell, 2018). It is suggested that CBT may benefit from working on children’s empathic abilities, in particular on feeling with the other or sharing another’s emotional experience (i.e., affective empathy or the emotional component of empathy), understanding of the other’s emotion (i.e., cognitive empathy or the cognitive component of empathy), and urge to taking care of another (i.e., sympathy or the motivational component of empathy) (Decety & Cowell, 2014; Decety & Jackson, 2004; Spinrad et al., 2023). Learning to distinguish between the three components of empathy is crucial. The motivational component is especially important with a view to deploying prosocial behavior. Children with conduct problems, perhaps specifically those with limited prosocial emotions, must learn to pay attention to the child’s distress towards whom they start displaying aggressive behavior and must experience themselves how it feels like if this is done to them, with the aim of stopping this behavior. This requires a lot of practice, perhaps adding virtual reality, as individuals tend to respond realistically to virtual simulations of real-life events (Dellazizzo et al., 2019).

In *step 6* children learn setting goals which may build the bridge between the complex interpretation step and the generation of solutions. Goals function as orientations toward particular outcomes and therefore are thought to influence subsequent response generation (Crick & Dodge, 1994). Moral values such as high values for dominance, revenge and self-enhancement, and low values for affiliation may affect goal orientations (Crick & Dodge, 1996; Lochman et al., 1993). In CBT, therapists need to work with children on setting relationship-enhancing, affiliation goals rather than dominance and revenge goals, in view of generating appropriate solutions (step 7). Also, difficulty to regulate angry and anxious emotions may endorse revenge goals (McDonald & Lochman, 2012) which involves working on emotion regulation (step 3) as well.

In *step 7* children learn generating solutions. In CBT, children are typically encouraged to come up with as many solutions as possible which then are categorized into solution types such as verbal assertion, compromise, conciliation, help-seeking, verbal aggression, and physical aggression. Normative beliefs in support of aggression and antisocial behavior may yield a bias to aggressive and antisocial responses to social problems (Huesmann & Guerra, 1997; Zelli et al., 1999). Therefore, changing these beliefs into beliefs in support of prosocial solutions is needed here. At the same time, children learn to come up with solutions that bring both the other person and themselves benefits.

Inappropriate solutions may result from atypical social problem-solving steps, including hostile interpretations and the conviction that this is related to how people treat each other, deviant norms concerning actions that can harm others physically and psychologically, difficulty with empathy, and setting goals of dominance and revenge. Therefore, making connections between all preceding social problem-solving steps and appropriate solutions is needed in CBT. These include adequate interpretations, normative beliefs about prosocial solutions, appropriate care-based norms, empathy, and high goal values for affiliation.

*Step 8* is about the evaluation of possible solutions based on outcome expectations and normative beliefs. After children have come up with solutions the therapist can ask questions about the consequences of these solutions and about the moral acceptability of the solutions: ‘What do you think will happen if you do or say that? Will that help solve the problem? What is the direct effect for yourself and for the other? And what is the effect in a week or a month? Do I not harm the other person with this solution? Is it correct to do that?”.

Aggressive children expect aggressive behavior to lead to favorable outcomes for they have learned that aggression reduces aversive treatment by other people (see principle of negative reinforcement and Patterson’s Coercive Theory, 1982). Indeed, aggressive children are more confident that aggression will produce tangible rewards and will reduce aversive treatment by others compared to non-aggressive children (Perry et al., 1986). Children with conduct problems, therefore, should actually experience that appropriate behaviors result in positive consequences on the short and long term for both themselves, the other person, and their relationship. Therefore, in addition to work with children on these themes, therapists in their work with parents and other adults teach them how to elicit and then reinforce appropriate behavioral responses in the child. Subsequently, these children’s experiences with appropriate behaviors and their positive outcomes are shared and discussed in CBT.

In addition, on the basis of their normative beliefs about aggression, children with conduct problems are less likely to reject aggressive solutions once they have thought of them as solutions to social problems (Huesmann & Guerra, 1997; Zelli et al., 1999). They worry less about not harming other persons (Blair, 2007, 2017). Normative beliefs about aggression, including social acceptability and moral appropriateness of aggression, will change when children with conduct problems experience for themselves that socially appropriate behaviors “work”, in that they result in positive outcomes both for the other person and themselves, on the short and the long term.

The final *step 9* is about decision-making. Cognitive neuroscience studies in children with conduct problems show difficulties in decision-making; uncertainties about positive and negative outcomes can impede these children’s and adolescents’ ability to make decisions about appropriate solutions to social problems (Blair, 2017; Blair et al., 2018). In line with this, children with conduct problems more often selected an aggressive response among various responses shown in videos even after an extensive assessment of social problem-solving in which examples of appropriate responses were shown and numerous questions about the responses asked (Matthys et al., 1999; Van Rest et al., 2020). Thus, in CBT making connections between all preceding social problem-solving steps and appropriate solutions is highly needed to improve children’s decision-making. However, many positive experiences with appropriate solutions are needed to increase their likelihood to decide to use these solutions in everyday life. In addition, these appropriate (cognitive) solutions must be expressed in appropriate behaviors (i.e., social skills). Thus, role-plays are warranted to expand children’s behavioral repertoire.

## Discussion and Conclusion

Moral thinking and empathy are not often considered relevant themes in CBT programs for the treatment of conduct problems. Our suggestions about including moral thinking and empathy in CBT for conduct problems are based on current insights into morality from research in developmental psychology and cognitive neuroscience. Moreover, we have attempted to integrate moral thinking and empathy into social problem-solving skills. For example, in view of generating appropriate solutions to social problems normative beliefs in support of prosocial solutions, appropriate care-based norms, empathy, and high goal values for affiliation are considered. The integration of moral themes into social problem-solving and the translation of these themes in terms of their functional meaning for appropriate behavior can aid children with conduct problems to see the usefulness of morality. Importantly, children with conduct problems not only differ in the type of characteristic behaviors and associated problems, but also in social problem-solving skills, as well as in moral thinking and empathy. This heterogeneity is essential from the perspective of achieving changes in social problem-solving and moral functioning through discussions and sharing of new experiences in the course of the psychological treatment.

CBT, therefore, needs to be tailored to target the child’s impaired psychological functions. The latter may differ depending not only on the characteristic symptoms of conduct problems, but also on symptoms of associated conditions (e.g., attention problems, impulsivity, anxiety). Associations between conduct problems and symptoms of attention-deficit/hyperactivity disorder are especially relevant to consider as attention-deficit/hyperactivity disorder and oppositional defiant disorder or conduct disorder often co-occur (Angold et al., 1999). In this context, additional pharmacological treatment of severe symptoms of attention-deficit/hyperactivity disorder may be needed (e.g., methylphenidate, atomoxetine) (National Institute for Health & Care Excellence, 2018).

Psychotherapists may also want to take into consideration differences in the cognitive and language developmental level of participating children and adolescents. Relatedly, although the various psychological functions targeted in CBT are assumed to be developed in children as young as 7 years old, albeit on a simple level, the interplay between those functions is more difficult for 7 to 8 year old children to grasp than for 10–11 year old children and for adolescents. In addition, there are age-related changes in morality, for example, children mainly focus on avoidance of harm and benefits to others through actions of helping and sharing, whereas adolescents also maintain concepts of justice and rights (Turiel, 2023). However, psychotherapists take age into account when forming groups of children and adolescents.

Concerns have arisen about delivering interventions for children and adolescents with conduct problems in group formats. Dishion and colleagues (1999) examined potential deviant peer effects in the context of a cognitive-behavioral group intervention for adolescents. At 1-year follow-up, adolescents who had received youth sessions (youth-only group) had higher rates of tobacco use and of teacher-rated delinquent behaviors than did control children (parent-only group, youth and parent combined group, attention placebo group). However, meta-analyses have not found consistent evidence for deviancy training effects within group interventions for youth with conduct problems (e.g., Weiss et al., 2005). Still, concerns about deviancy training (i.e., during group sessions deviant peers reinforce each other’s antisocial actions and words) remain and may be addressed as follows (Matthys & Lochman, 2017). At the stage of composing a group, some group members serve as solid peer models for how to enact more competent, verbal assertion and negotiation strategies. During the group sessions, enhancing a positive group process is achieved by including positive feedback time from all group members at the end of group sessions. When disagreements and conflicts develop between group members during sessions, these can be opportunities to directly model and reinforce the social problem-solving skills which are the focus of the interventions (Matthys & Lochman, 2017).

One may question whether moral functioning in children with conduct problems can be changed at all. The Fast Track study showed that a multiyear indicated preventive intervention offered at schools including not only the promotion of children’s social-cognitive and social skills but also the improvement of parenting skills and academic mentoring, resulted in a decrease of antisocial behavior. This reduction was mediated by its impact on three social-cognitive processes: (1) Reducing hostile-attribution biases, (2) increasing the generation of socially competent responses to social problems, and (3) improving the evaluation of the outcomes of aggression as detrimental (i.e., devaluing aggression as effective and acceptable) (Dodge et al., 2013). This study not only demonstrates that devaluing aggression as effective and acceptable (i.e., a normative belief) is feasible, but is also a mechanism of change and as such constitutes an important aspect of cognitive-behavioral oriented treatment approaches. It should be noted that changes were achieved not only by influencing children’s cognitions, but also by improving their social skills and by working with parents on their parenting skills. In this context, it should be added that working on moral values with parents may be needed as well. If youths’ changing moral values are not supported by their parents and siblings, there is a risk that these changes will only be temporary. There is also evidence that school-based interventions to promote empathy-related responding have a small but positive effect on conduct problems (*d* = 0.17) (Malti et al., 2016).

The learning processes to change social problem-solving skills in children with conduct problems, including moral topics such as devaluing aggression as effective and acceptable, need to be intensive and lengthy, though we do not know for how long. Of course CBT cannot be expected to last for years, but treatment could be intensified by involving parents, child care workers, and teachers in CBT in view of allowing the learning processes to take place in everyday life. After termination of CBT, parents and teachers must continue to support the child’s learning processes.

In conclusion, moral thinking and empathy can be part of CBT for conduct problems. A functional integration of moral thinking and empathy into social problem-solving can promote the acceptance of moral topics by children with conduct problems. Here we offered suggestions how to include morality and empathy in CBT. Psychotherapists can use these suggestions in their clinical work with children, their parents and other adults. In addition, extant programs can be adapted accordingly.

## Data Availability

Data sharing not applicable to this article as no datasets were generated or analysed during the review.
